# Instant Pulmonary Embolus and Deep Vein Thrombosis Diagnosis Through Point-of-Care Ultrasound in a Patient Diagnosed With Sepsis Due to a Urinary Tract Infection

**DOI:** 10.7759/cureus.45790

**Published:** 2023-09-22

**Authors:** Larry Istrail

**Affiliations:** 1 Hospital Medicine, Inova Fairfax Hospital, Falls Church, USA

**Keywords:** point-of-care ultrasound, point-of-care ultrasound (pocus), critical echo and ultrasonography, pulmonary critical care, pulmonary disease, 2d echo

## Abstract

Pulmonary embolus (PE) is a life-threatening illness that can be fatal if not treated with anticoagulation in a timely fashion. Without the use of CT angiography for direct visualization of thrombus in the pulmonary arteries or downstream vessels, the diagnosis of a PE can be challenging. Point-of-care ultrasound (POCUS) is a bedside method that is highly effective in directly evaluating deep vein thrombosis (DVT) and indirectly evaluating PE. In this case, a patient presented to the hospital with hypotension from a presumed urinary tract infection and was treated with intravenous fluids and antibiotics. POCUS examination revealed significant right heart strain and femoral DVT despite negative imaging one week prior. This highlights the importance of POCUS in evaluating patients with cardiopulmonary disease.

## Introduction

Pulmonary embolus (PE) is a disease that can be difficult to diagnose and can lead to significant morbidity and mortality if not treated with anticoagulation in a timely fashion. Without the use of CT angiography (CTA) for direct visualization of thrombus in the pulmonary arteries or downstream vessels, the diagnosis of a PE can be a challenging one. Clinical suspicion scores and laboratory tests such as a D-dimer can be used for the indirect assessment of PE and deep vein thrombosis (DVT), though their clinical usefulness is limited. The Wells’ criteria is a set of objective and subjective clinical findings that are associated with a DVT that are often utilized. These criteria include leg swelling, a history of DVT, recent surgery or leg immobilization, hemoptysis, or a history of malignancy. However, even if all criteria are met, the incidence of PE was only 28-40% in the outpatient setting [1]. In the inpatient setting, Wells’ criteria only performed slightly better than chance for determining if a DVT was present [2]. The Wells’ criteria are further weakened by the fact that they were validated against lower sensitivity ventilation and perfusion scans, which would artificially inflate the diagnostic accuracy [3]. The D-dimer is a degradation product of a cross-linked fibrin blood clot. As D-dimer can be elevated for many other reasons besides clotting, it is not specific to DVT or PE. However, it is 95-99% sensitive [4] and can effectively rule out a DVT or PE if the D-dimer is normal. Yet, its low specificity limits its usefulness if it is moderately elevated or high.

Point-of-care ultrasound (POCUS) is another bedside method for evaluating DVT or PE. Clinician-performed compression examinations at the bedside can directly diagnose DVT with a high degree of accuracy equivalent to formal Doppler studies [5,6]. Directly diagnosing a PE with POCUS is usually not possible; however, there are multiple indirect signs that increase the likelihood. With cardiac POCUS, evidence of right ventricular dilation, right ventricular wall hypokinesis with apical sparing (McConnel’s sign), and right ventricular outflow tract Doppler increased acceleration time and early systolic notching are all echocardiographic signs that can be acquired with POCUS that have low sensitivity and relatively high specificity for PE [7-9]. When multiple POCUS findings are combined in a comprehensive bedside POCUS examination, sensitivity can approach that of CTA. When compared to CTA, in patients with elevated Wells’ score or D-dimer, multiorgan POCUS of the heart, lungs, and lower extremities was 90% sensitive and 86% specific for diagnosing a PE [10].

## Case presentation

An 87-year-old female with a history of hypertension presented to the hospital with hypotension and altered mental status. One week before admission, she had been hospitalized after a mechanical fall. Imaging of her hip was negative for acute fracture. She was also found to have mild hypoxic respiratory failure for which she was treated with intravenous (IV) diuretics. She underwent an echocardiogram which was normal, as well as a lower extremity Doppler study which was negative for DVT in both legs. On the day of admission, she was found to be acutely hypotensive at her rehab center and was brought to the hospital. She had undergone a urinalysis and urine culture three days before arrival at the hospital which grew 25-50,000 CFU/mL of *Proteus *and over 100,000 CFU/mL of Klebsiella, both sensitive to ceftriaxone.

On arrival at the emergency department, she was hypotensive with blood pressure as low as 70/42 mmHg. Her heart rate was 64-69 breaths/minute, respiratory rate was 20-30 breaths/minute, and oxygen saturation was 98% on 2 L of oxygen via a nasal cannula. Her labs were significant for a leukocytosis of 16,900 white blood cells, acute kidney injury with a creatinine of 2.2 mg/dL, lactic acid of 2.1 mmol/L, and urinalysis with large leukocyte esterase, moderate blood, and too numerous to count white blood cells. A chest X-ray showed chronic increased interstitial markings, and an X-ray of the right hip showed a mildly displaced fracture of the lesser trochanter, for which orthopedics did not recommend surgical intervention. She was treated with 3 L of normal saline and started on IV ceftriaxone. Her blood pressure improved and she was transferred to the general medicine team. The following morning, the patient was placed on 6 L of oxygen through her nasal cannula with an oxygen saturation of 97%. Labs were significant for an improved leukocytosis with a white blood cell count of 12,000 and improved but still abnormal renal function with a creatinine of 1.9 mg/dL.

A thorough cardiopulmonary and venous POCUS was performed by this author. With the head of the bed at 45 degrees, her right internal jugular vein examination revealed a distended vein with absent pulsations consistent with jugular venous distention (Video 1).

**Video 1:** Jugular venous distention visualized with point-of-care ultrasound. With the head of the bed at 45 degrees, the right internal jugular vein is distended with absent pulsations consistent with jugular venous distention.

The jugular vein collapse point (wine bottle sign) was seen 2 cm below the mandible, consistent with elevated right-sided pressures. A lung examination showed lung sliding of smooth, regular-appearing pleura with diffuse B-lines in multiple rib spaces, consistent with interstitial syndrome due to pulmonary edema (Video 2).

**Video 2:** Interstitial syndrome with regular pleura and multiple B-lines consistent with pulmonary edema. Anterior lung surface with lung sliding, regular pleura, and multiple B-lines consistent with pulmonary edema.

A cardiac examination revealed normal left ventricular function in the parasternal long-axis view (Video 3).

**Video 3:** Right ventricular dilation seen in parasternal long-axis view. This parasternal long-axis view of the heart reveals a normal left ventricular ejection fraction with right ventricular dilation.

The parasternal short-axis view confirmed an enlarged right ventricle with flattening of the septum consistent with elevated right-sided pressure (Video 4).

**Video 4:** Right ventricular dilation seen in parasternal short-axis view. In the parasternal short-axis view, the right ventricle is larger than the left ventricle. There is also flattening of the septum which created the “d-sign” in the left ventricle consistent with elevated right-sided pressure.

The apical four-chamber view revealed McConnell’s sign, a dilated right ventricle with reduced wall motion and apical sparing (Video 5).

**Video 5:** Apical four-chamber view showing McConnell’s sign. In the apical four-chamber view, the right ventricle is dilated and the wall motion is limited, except for the apex which is consistent with McConnell’s sign, which is specific but not sensitive for pulmonary embolus.

With new evidence of right heart strain compared to her normal comprehensive echocardiogram one week prior, a PE was suspected. A bedside lower extremity compression examination was completed to evaluate for DVT, which discovered a non-compressible left femoral vein consistent with a DVT that was not present one week prior (Video 6).

**Video 6:** Left common femoral vein compression examination with deep vein thrombosis present. The femoral vein (right lower vessel) does not compress with ultrasound probe pressure due to the presence of deep vein thrombosis. The femoral artery is seen above to the left.

**Figure 1:**
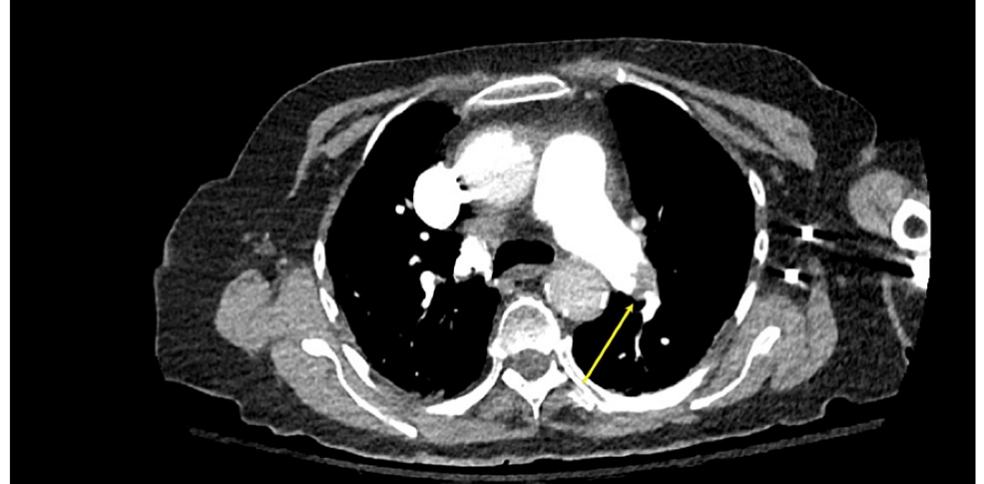
CT angiogram showing thrombus in the left pulmonary artery. Thrombus is seen here in the left pulmonary artery, confirming the diagnosis of a pulmonary embolus.

Out of concern for the patient’s renal function and possible contrast-induced nephropathy, CTA was deferred and a STAT D-dimer was ordered and found to be very elevated at 26.13 µg/mL, and she was started on IV anticoagulation for the treatment of DVT and presumed PE, as well as IV diuretics for the treatment of her pulmonary edema and venous congestion. A formal lower extremity Doppler study confirmed a new occlusive thrombus in the left femoral vein, and a formal echocardiogram confirmed the POCUS findings of acute right heart strain and moderate pulmonary hypertension. She diuresed well and her oxygen was slowly weaned. Her kidney function improved the following day and a CTA was ordered which revealed PE in bilateral main pulmonary arteries extending into the lobar and segmental branches with high clot burden and right heart strain (Figure 1).

## Discussion

DVT and PE are causes of hypotension and hypoxia that must be considered in any hospitalized patient. This patient presented with shortness of breath, altered mental status, and abnormal urinalysis, and was admitted to the hospital and treated for sepsis due to a urinary tract infection. Her urinalysis was abnormal; however, her prior urinalyses were abnormal as well, suggesting she may have been colonized and not actively infected. Given her immobility from her fall and hip fracture, she was at high risk for developing a DVT. Yet, with a normal echocardiogram and normal lower extremity Doppler examinations one week prior, it is unlikely that her hypotension and hypoxic respiratory failure were being caused by a PE, and therefore this diagnosis was not considered on admission.

Without POCUS to perform a thorough cardiopulmonary and venous examination at the bedside, this may have not been detected at all. POCUS allows the clinician to overcome anchoring bias and evaluate alternative diagnoses at the bedside in real time. This delay in correct diagnosis may also have resulted in significant harm, as she received 3 L of intravenous fluids for her hypotension, worsening her pulmonary edema and hypoxic respiratory failure. While POCUS usually cannot diagnose a PE directly, it can be used to rule in or out a DVT with near certainty, as well as to accurately assess what effect a potential thrombus is having on the right heart. This combined with a D-dimer is a powerful way to evaluate patients with hypoxic respiratory failure suspected to be caused by a PE.

## Conclusions

As demonstrated here, POCUS examinations can drastically alter a patient’s clinical course. When evaluating a patient with shortness of breath, when PE is suspected, a lower extremity venous Doppler examination can directly diagnose or rule out a DVT. Cardiac POCUS can evaluate for evidence of right heart strain to provide indirect evidence of a pulmonary embolus. These POCUS examinations can augment clinical suspicion and interpretation of D-dimer laboratory findings to diagnose PE more effectively without the use of CTA.
